# Urinary incontinence in Ugandan gynecologic outpatient clinics: Prevalence, clinical types, and associated factors

**DOI:** 10.1371/journal.pgph.0007221

**Published:** 2026-09-15

**Authors:** John Katongole, Emmanuel Okurut, Daniel Udofia Owu, Theoneste Hakizimana, Prosper Akankwasa, Ester Namutosi, Musa Kasujja

**Affiliations:** 1 Department of Obstetrics and Gynecology, Kampala International University, Bushenyi, Uganda; 2 Department of Physiology, Kampala International University, Bushenyi, Uganda; China Medical University College of Public Health, CHINA

## Abstract

Urinary incontinence (UI) is a common but underrecognized condition among women, including within routine gynecologic outpatient care, particularly in low- and middle-income countries. Evidence from referral-level gynecologic outpatient settings in Uganda remains limited. This study therefore determined the prevalence, clinical types, and associated factors of UI among women attending gynecologic outpatient clinics in Uganda. A cross-sectional study was conducted among 374 non-pregnant women attending gynecologic outpatient clinics at Kayunga and Jinja Regional Referral Hospitals (RRH). UI and its clinical types were assessed using the Questionnaire for Urinary Incontinence Diagnosis (QUID). Data on associated factors were collected using structured interviews, and multivariable logistic regression was used to identify factors independently associated with UI. Among the 374 women enrolled, 103 had UI, giving a prevalence of 27.5% (95% confidence interval [CI]: 23.1 to 32.3). The most common subtype was stress UI (44.7%), followed by urgency UI (30.1%) and mixed UI (25.2%). Increasing age was independently associated with UI, with higher odds among women aged 30–39 years (adjusted odds ratio [AOR] 2.03, 95% CI 1.00 to 4.10), 40–49 years (AOR 2.63, 95% CI 1.18 to 5.88), and 50 years or older (AOR 4.95, 95% CI 1.25 to 19.56) compared with women younger than 30 years. No formal education (AOR 4.34, 95% CI 1.29 to 14.55), high parity of four or more deliveries (AOR 2.97, 95% CI 1.21 to 7.26), and prolonged labor lasting more than 24 hours (AOR 2.46, 95% CI 1.37 to 4.40) were also independently associated with UI. Urinary incontinence affected approximately one in four women attending gynecologic outpatient clinics in Uganda, with stress UI as the predominant subtype. Routine UI screening in gynecologic outpatient services and strengthened obstetric and postpartum care may help reduce the burden of UI in similar referral settings.

## Introduction

Urinary incontinence (UI), the involuntary loss of urine, is a common and distressing condition that affects women throughout life [1]. Although it is not life threatening, it is associated with important physical, psychosocial, sexual, and economic consequences, including reduced quality of life, social withdrawal, reduced work productivity, and increased healthcare use [2,3]. In many low- and middle-income settings, UI remains underrecognized and undertreated because women may not volunteer symptoms unless they are directly asked.

Globally, the prevalence of UI among women varies widely, from approximately 5% to more than 45%, depending on age, population characteristics, case definitions, and diagnostic approaches [3,4]. Prevalence increases from about 10% to 15% in younger adult women to more than 40% in older age groups, and clinic-based samples often report higher burdens than community surveys because they concentrate symptomatic and care-seeking women [3,5,6].

Stress UI is typically the most common clinical subtype, followed by urgency UI and mixed UI, although subtype distributions vary by reproductive history, comorbidity burden, and care setting [5,7]. Stress UI usually reflects pelvic floor weakness or urethral hypermobility during rises in intra-abdominal pressure, urgency UI is more closely related to urgency and detrusor overactivity, and mixed UI combines features of both [8].

In sub-Saharan Africa, available evidence indicates that UI affects a substantial proportion of women seeking care, but the evidence base remains limited and heterogeneous. Clinic-based studies have reported prevalence estimates of about 12% in Ghana and 17% to 30% in other African settings, while pregnancy and postpartum studies often report even higher prevalence [9–11]. Stigma, normalization of leakage after childbirth or with aging, limited pelvic floor health services, and competing priorities within resource-constrained health systems can delay disclosure, diagnosis, and treatment.

In Uganda, evidence on UI is particularly sparse outside specialized or high-risk settings. Existing studies have largely focused on women with severe pelvic floor compromise, such as persistent incontinence after obstetric fistula repair, or on antenatal and postpartum populations, limiting generalizability to women attending routine gynecologic outpatient clinics [10,12,13].

Understanding the prevalence, clinical types, and associated factors of UI within referral-level gynecologic outpatient settings is essential for informing clinical practice and health system planning. Clinic-based estimates can guide routine screening, identify women at highest risk, and support the integration of pelvic floor health (PFH) into gynecologic and maternal health services, particularly in resource-limited settings [2,14].

This cross-sectional study therefore determined the prevalence of urinary incontinence, described its clinical types, and identified factors associated with UI among women attending gynecologic outpatient clinics at Kayunga and Jinja Regional Referral Hospitals (RRH) in Uganda. Compared with prior studies from sub-Saharan Africa, this study adds referral-level Ugandan evidence from routine gynecologic outpatient clinics, using the QUID to estimate UI prevalence, describe clinical subtypes, and identify associated sociodemographic, obstetric, and clinical factors among non-pregnant women. This setting is important because referral-level gynecologic clinics are a practical but understudied point of contact for identifying cumulative or unresolved pelvic floor morbidity. The findings provide context-specific evidence to inform clinical care, prevention strategies, and future research in similar low-resource settings.

## Methods

### Study design and setting

This hospital-based cross-sectional study was conducted among women attending gynecologic outpatient clinics at Kayunga and Jinja Regional Referral Hospitals (RRH) in Uganda from 10th July to 8th October, 2025. Both hospitals are public regional referral facilities that provide specialist obstetric and gynecologic services to predominantly rural and peri-urban populations in central and eastern Uganda, respectively.

### Study population and eligibility criteria

The source population comprised all non-pregnant women attending gynecologic outpatient clinics at Kayunga and Jinja Regional Referral Hospitals (RRH) during the study period. Eligible participants were women aged 18–55 years attending with any gynecologic complaint and willing to provide informed consent. The age range was selected to focus on reproductive and early post-reproductive women commonly managed in these clinics while limiting confounding from frailty-related incontinence at advanced ages. Women who were currently pregnant, within six weeks postpartum, critically ill, unable to complete an interviewer-administered questionnaire, or who declined consent were excluded.

### Sample size and sampling

The sample size determination was aligned with the study objectives. For prevalence estimation, the Kish and Leslie single population proportion formula, n = Z^2^p(1-p)/d^2^, was applied, where Z = 1.96 for 95% confidence, p = 0.67 based on a prior Ugandan high risk population study [12], and d = 0.05. This yielded a minimum sample size of 340 participants. Allowing for 10% nonresponse or incomplete data, the final target sample size was 374 participants, which was considered adequate for prevalence estimation and multivariable analysis.

Consecutive sampling was performed. All eligible women who attended during the study period were approached until the target sample size was reached. Of the 419 women assessed for eligibility, 374 consented to participate in the study.

### Study procedures and data collection

After screening for eligibility and obtaining written informed consent, participants were interviewed by trained research assistants using structured questionnaires in a private clinic setting. Recruitment, consent, questionnaire administration, and QUID screening were completed during the same outpatient encounter.

Urinary incontinence screening and classification were performed using the Questionnaire for Urinary Incontinence Diagnosis (QUID), a validated six-item instrument comprising three stress-related and three urgency-related symptom questions [15]. Responses were recorded on a frequency scale and summed to generate stress and urgency subscale scores. The QUID scoring thresholds used for classification are described below, and the scoring guide is listed as S1 Appendix in the supporting information captions.

### Variables

The primary outcome was urinary incontinence status (present or absent), defined as a positive QUID screen. Stress UI was diagnosed with a stress subscale score of ≥4 and an urgency subscale score of <6. Urgency UI was diagnosed when the urgency subscale score was ≥ 6 and a stress subscale score  < 4. Mixed UI was defined as stress subscale scores ≥4 and urgency subscale scores ≥6.

The independent variables were age, education level, marital status, parity, mode of delivery, and history of prolonged labor. Parity was defined as the number of prior births at 28 weeks of gestation or more. History of prolonged labor was operationalized as any previous labor reported to have lasted more than 24 hours, irrespective of parity, because detailed partograph records from previous deliveries were not consistently available. Body mass index (BMI) was calculated from measured or recorded weight and height in kg/m^2^ and categorized using standard thresholds. Hypertension and diabetes mellitus were based on self-report of a prior clinician diagnosis because complete historical case records were not uniformly available in the outpatient setting; these variables were treated as background covariates and interpreted cautiously.

### Statistical analysis

Data were analyzed using Stata version 17. Descriptive statistics were used to summarize participant characteristics and urinary incontinence prevalence with 95% confidence intervals. Bivariate logistic regression was used to assess associations between independent variables and UI. Variables with p < 0.20 at bivariate analysis were entered into the multivariable logistic regression model to avoid premature exclusion of potentially important confounders. Adjusted odds ratios with 95% confidence intervals were reported, with statistical significance set at p < 0.05. Model diagnostics included assessment of multicollinearity using variance inflation factors and review of model classification performance, and model estimates were interpreted with attention to precision and clinical plausibility. Analyses were based on complete-case data.

### Ethical considerations

Ethical approval was obtained from the Kampala International University Research Ethics Committee (approval number KIU-2025–1691). The study was conducted in accordance with the Declaration of Helsinki. Written informed consent was obtained from all participants. Participation was voluntary, and participants could withdraw at any time without affecting their clinical care. Confidentiality was maintained using unique identifiers and secure data handling accessible only to the research team.

## Results

### Participant flow and sample characteristics

**Fig 1:**
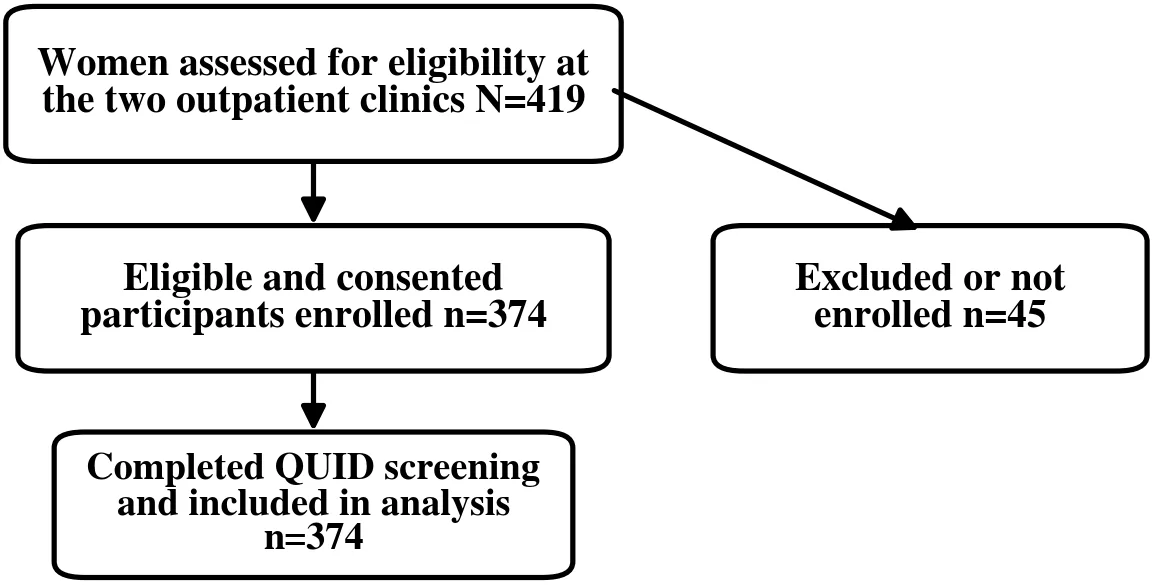
Participant flow diagram.

A total of 419 women were assessed for eligibility, of whom 374 consented and were enrolled in the study. All enrolled participants completed QUID screening and were included in the analysis. The participant flow is presented in Fig 1.

### Descriptive characteristics of the study population

The characteristics of the study population are summarized in Table 1, Table 2, Table 3. The median age of participants was 32 years, with an interquartile range of 26–39 years. Most participants were married, had attained primary or secondary education, and were multiparous. Vaginal delivery was the most commonly reported delivery mode, and about one third of participants had a body mass index (BMI) of 25 kg/m^2^ or higher.

**Table 1:** Sociodemographic and behavioral characteristics of study participants (N = 374).

Characteristic	Kayunga RRH n (%)	Jinja RRH n (%)	Overall n (%)	χ²(df), p-value
**Age (years)**				3.885(3), 0.274
<30	38 (23.0)	48 (23.0)	86 (23.0)	
30-39	86 (52.1)	106 (50.7)	192 (51.3)	
40-49	39 (23.6)	45 (21.5)	84 (22.5)	
50+	2 (1.2)	10 (4.8)	12 (3.2)	
**Marital status**				1.579(1), 0.209
Married	116 (70.3)	159 (76.1)	275 (73.5)	
Not-married	49 (29.7)	50 (23.9)	99 (26.5)	
**Place of residence**				1.776(1), 0.183
Rural	93 (56.4)	132 (63.2)	225 (60.2)	
Urban	72 (43.6)	77 (36.8)	149 (39.8)	
**Level of education**				8.602(3), 0.035
No formal education	15 (9.1)	13 (6.2)	28 (7.5)	
Secondary	69 (41.8)	71 (34.0)	140 (37.4)	
Primary	74 (44.9)	101 (48.3)	175 (46.8)	
Tertiary	7 (4.2)	24 (11.5)	31 (8.3)	
**Occupation**				0.455(2), 0.797
Formal employment	39 (23.6)	53 (25.4)	92 (24.6)	
Unemployed	50 (30.3)	67 (32.1)	117 (31.3)	
Non formal employment	76 (46.1)	89 (42.6)	165 (44.1)	
**Monthly household income**				1.575(2), 0.455
<200,000 UGX	60 (36.4)	67 (32.1)	127 (34.0)	
200,000–400,000 UGX	63 (38.2)	77 (36.8)	140 (37.4)	
>400,000 UGX	42 (25.5)	65 (31.1)	107 (28.6)	
**History of smoking**				0.589(1), 0.443
No	155 (93.9)	200 (95.7)	355 (94.9)	
Yes	10 (6.1)	9 (4.3)	19 (5.1)	
**Regular physical activity (≥3 days/week)**				0.001(1), 0.970
No	99 (60.0)	125 (59.8)	224 (59.9)	
Yes	66 (40.0)	84 (40.2)	150 (40.1)	
**Ever use of contraception**				0.229(1), 0.633
No	78 (47.3)	104 (49.8)	182 (48.7)	
Yes	87 (52.7)	105 (50.2)	192 (51.3)	

Note: The median age of the overall sample was 32 years (IQR: 26–39). UGX, Ugandan shillings.

**Table 2:** Reproductive characteristics of study participants (N = 374).

Characteristic	Kayunga RRH n (%)	Jinja RRH n (%)	Overall n (%)	χ²(df), p-value
**Parity**				4.420(4), 0.352
0	29 (17.6)	33 (15.8)	62 (16.6)	
1	33 (20.0)	42 (20.1)	75 (20.1)	
2	47 (28.5)	47 (22.5)	94 (25.1)	
3	33 (20.0)	42 (20.1)	75 (20.1)	
4+	23 (13.9)	45 (21.5)	68 (18.2)	
**History of macrosomic baby (>4 kg)**				0.056(1), 0.813
No	131 (79.4)	168 (80.4)	299 (80.0)	
Yes	34 (20.6)	41 (19.6)	75 (20.0)	
**Mode of last delivery**				0.345(1), 0.557
Vaginal	129 (78.2)	158 (75.6)	287 (76.7)	
Cesarean	36 (21.8)	51 (24.4)	87 (23.3)	
**Place of last delivery**				2.152(1), 0.142
Health facility	143 (86.7)	191 (91.4)	334 (89.3)	
Home	22 (13.3)	18 (8.6)	40 (10.7)	
**History of prolonged labor (>24 hours)**				0.211(1), 0.646
No	127 (77.0)	165 (79.0)	292 (78.1)	
Yes	38 (23.0)	44 (21.0)	82 (21.9)	

**Table 3:** Clinical and anthropometric characteristics of study participants (N = 374).

Characteristic	Kayunga RRH n (%)	Jinja RRH n (%)	Overall n (%)	χ²(df), p-value
Body mass index				1.123(3), 0.771
Underweight	18 (10.9)	17 (8.1)	35 (9.4)	
Normal	72 (43.6)	99 (47.4)	171 (45.7)	
Overweight	49 (29.7)	59 (28.2)	108 (28.9)	
Obese	26 (15.8)	34 (16.3)	60 (16.0)	
**Hypertension**				1.015(1), 0.314
No	142 (86.1)	187 (89.5)	329 (88.0)	
Yes	23 (13.9)	22 (10.5)	45 (12.0)	
**Diabetes mellitus**				1.541(1), 0.214
No	154 (93.3)	201 (96.2)	355 (94.9)	
Yes	11 (6.7)	8 (3.8)	19 (5.1)	
**Chronic cough**				3.448(1), 0.063
No	154 (93.3)	183 (87.6)	337 (90.1)	
Yes	11 (6.7)	26 (12.4)	37 (9.9)	
**Chronic constipation**				0.042(1), 0.837
No	141 (85.5)	177 (84.7)	318 (85.0)	
Yes	24 (14.5)	32 (15.3)	56 (15.0)	
**Neurological illness**				1.016(1), 0.313
No	158 (95.8)	204 (97.6)	362 (96.8)	
Yes	7 (4.2)	5 (2.4)	12 (3.2)	
**History of abdominal, pelvic, or spine surgery**				0.322(1), 0.570
No	142 (86.1)	184 (88.0)	326 (87.2)	
Yes	23 (13.9)	25 (12.0)	48 (12.8)	

### Prevalence of urinary incontinence

Overall, UI was identified in 103 of 374 participants, giving a prevalence of 27.5% (95% confidence interval: 23.1 to 32.3). Prevalence was comparable across the two study sites. The distribution of UI status is shown in Table 4.

**Table 4:** Prevalence of urinary incontinence by study site (N = 374).

UI status	Kayunga RRH n (%) [95% CI]	Jinja RRH n (%) [95% CI]	Overall n (%) [95% CI]	χ²(df), p-value
No UI	115 (69.7) [62.2-76.3]	156 (74.6) [68.3-80.1]	271 (72.5) [67.7-76.8]	1.130(1), 0.288
UI	50 (30.3) [23.7-37.8]	53 (25.4) [19.9-31.7]	103 (27.5) [23.2-32.3]	

### Clinical types of urinary incontinence

**Fig 2:**
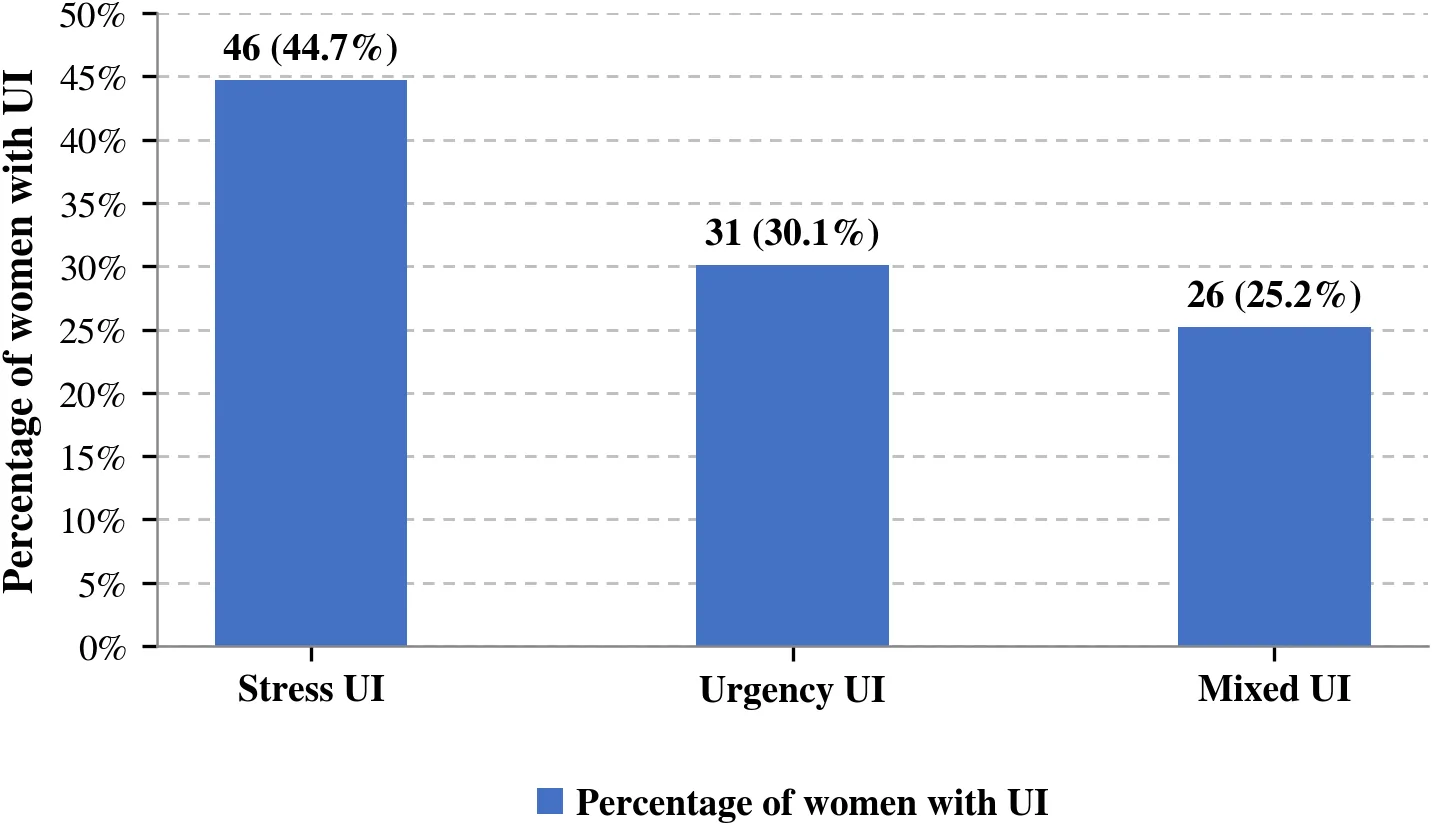
Distribution of clinical types of urinary incontinence among women with UI (N = 103).

Among women with UI, stress UI was the most frequent clinical type, accounting for 44.7% of cases. Urgency UI accounted for 30.1%, while mixed UI accounted for 25.2%. The distribution of clinical types is shown in Fig 2.

### Factors associated with urinary incontinence

In bivariate analysis, several participant, obstetric, and clinical characteristics were associated with UI (Table 5). Table 5 also presents subgroup-specific UI prevalence to facilitate comparison across participant characteristics. Variables meeting the prespecified inclusion threshold were entered into the multivariable logistic regression model.

**Table 5:** Subgroup prevalence and bivariate logistic regression of factors associated with UI (N = 374).

Characteristic	UI absent n (%)	UI present n (%)	Crude OR	95% CI	p-value
**Study site**					
Jinja RRH	156 (74.6)	53 (25.4)	1.00		
Kayunga RRH	115 (69.7)	50 (30.3)	1.28	0.81-2.02	0.288
**Age group**					
<30	72 (83.7)	14 (16.3)	1.00		
30-39	136 (70.8)	56 (29.2)	2.12	1.10-4.06	0.024*
40-49	57 (67.9)	27 (32.1)	2.44	1.17-5.07	0.017*
50+	6 (50.0)	6 (50.0)	5.14	1.45-18.28	0.011*
**Marital status**					
Married	198 (72.0)	77 (28.0)	1.00		
Not-married	73 (73.7)	26 (26.3)	0.92	0.54-1.54	0.740
**Place of residence**					
Urban	108 (72.5)	41 (27.5)	1.00		
Rural	163 (72.4)	62 (27.6)	1.00	0.63-1.59	0.993
**Education level**					
Tertiary	21 (67.7)	10 (32.3)	1.00		
No formal education	11 (39.3)	17 (60.7)	3.25	1.11-9.45	0.031*
Secondary	104 (74.3)	36 (25.7)	0.73	0.31-1.69	0.458
Primary	135 (77.1)	40 (22.9)	0.62	0.27-1.43	0.263
**Occupation**					
Formal employment	74 (80.4)	18 (19.6)	1.00		
Unemployed	77 (65.8)	40 (34.2)	2.14	1.12-4.06	0.020*
Non formal employment	120 (72.7)	45 (27.3)	1.54	0.83-2.86	0.170*
**Monthly household income**					
>400,000 UGX	79 (73.8)	28 (26.2)	1.00		
<200,000 UGX	89 (70.1)	38 (29.9)	1.20	0.68-2.14	0.525
200,000–400,000 UGX	103 (73.6)	37 (26.4)	1.01	0.57-1.80	0.963
**History of smoking**					
No	259 (73.0)	96 (27.0)	1.00		
Yes	12 (63.2)	7 (36.8)	1.57	0.60-4.11	0.355
**Regular physical activity (≥3 days/week)**					
No	164 (73.2)	60 (26.8)	1.00		
Yes	107 (71.3)	43 (28.7)	1.10	0.69-1.74	0.690
**Parity category**					
0	49 (79.0)	13 (21.0)	1.00		
1	55 (73.3)	20 (26.7)	1.37	0.62-3.04	0.438
2	67 (71.3)	27 (28.7)	1.52	0.71-3.24	0.279
3	54 (72.0)	21 (28.0)	1.47	0.66-3.24	0.344
4+	46 (67.7)	22 (32.4)	1.80	0.81-3.99	0.146*
**History of macrosomic baby (>4 kg)**					
No	217 (72.6)	82 (27.4)	1.00		
Yes	54 (72.0)	21 (28.0)	1.03	0.59-1.81	0.921
**Mode of last delivery**					
Cesarean	64 (73.6)	23 (26.4)	1.00		
Vaginal	207 (72.1)	80 (27.9)	1.08	0.63-1.85	0.793
**Place of last delivery**					
Health facility	244 (73.1)	90 (27.0)	1.00		
Home	27 (67.5)	13 (32.5)	1.31	0.65-2.64	0.458
**History of prolonged labor (>24 hours)**					
No	224 (76.7)	68 (23.3)	1.00		
Yes	47 (57.3)	35 (42.7)	2.45	1.47-4.11	0.001*
**Ever used contraception**					
No	136 (74.7)	46 (25.3)	1.00		
Yes	135 (70.3)	57 (29.7)	1.25	0.79-1.97	0.340
**BMI category**					
Normal	125 (73.1)	46 (26.9)	1.00		
Underweight	30 (85.7)	5 (14.3)	0.45	0.17-1.24	0.123*
Overweight	78 (72.2)	30 (27.8)	1.05	0.61-1.79	0.873
Obese	38 (63.3)	22 (36.7)	1.57	0.84-2.94	0.155*
**Hypertension**					
No	244 (74.2)	85 (25.8)	1.00		
Yes	27 (60.0)	18 (40.0)	1.91	1.00-3.65	0.049*
**Diabetes mellitus**					
No	258 (72.7)	97 (27.3)	1.00		
Yes	13 (68.4)	6 (31.6)	1.23	0.45-3.32	0.686
**Chronic cough**					
No	248 (73.6)	89 (26.4)	1.00		
Yes	23 (62.2)	14 (37.8)	1.70	0.84-3.44	0.143*
**Chronic constipation**					
No	236 (74.2)	82 (25.8)	1.00		
Yes	35 (62.5)	21 (37.5)	1.73	0.95-3.14	0.073*
**Neurological illness**					
No	266 (73.5)	96 (26.5)	1.00		
Yes	5 (41.7)	7 (58.3)	3.88	1.20-12.51	0.023*
**History of abdominal, pelvic, or spine surgery**					
No	237 (72.7)	89 (27.3)	1.00		
Yes	34 (70.8)	14 (29.2)	1.10	0.56-2.14	0.787

Percentages are row percentages within each category. *p < 0.20 indicates variables/categories that met the screening criterion for inclusion in the multivariable model.

In multivariable analysis (Table 6), increasing age remained independently associated with UI, with women aged 30 years and above having higher odds than younger women. Having no formal education, parity of four or more deliveries, and a history of prolonged labor lasting more than 24 hours also remained independently associated with UI.

**Table 6:** Multivariable logistic regression of factors associated with UI (N = 374).

Characteristic	cOR	95% CI	p-value	aOR	95% CI	p-value
**Age group**						
<30	1.00			1.00		
30-39	2.12	1.10-4.06	0.024	2.03	1.00-4.10	0.050**
40-49	2.44	1.17-5.07	0.017	2.63	1.18-5.88	0.019**
50+	5.14	1.45-18.28	0.011	4.95	1.25-19.56	0.023**
**Education level**						
Tertiary	1.00			1.00		
No formal education	3.25	1.11-9.45	0.031	4.34	1.29-14.55	0.017**
Secondary	0.73	0.31-1.69	0.458	0.92	0.36-2.35	0.859
Primary	0.62	0.27-1.43	0.263	0.79	0.32-1.98	0.615
**Occupation**						
Formal employment	1.00			1.00		
Unemployed	2.14	1.12-4.06	0.020	1.99	0.98-4.02	0.055
Non formal employment	1.54	0.83-2.86	0.170	1.89	0.96-3.75	0.067
**Parity category**						
0	1.00			1.00		
1	1.37	0.62-3.04	0.438	1.51	0.63-3.62	0.354
2	1.52	0.71-3.24	0.279	1.23	0.53-2.86	0.637
3	1.47	0.66-3.24	0.344	1.89	0.77-4.61	0.163
4+	1.80	0.81-3.99	0.146	2.97	1.21-7.26	0.017**
**BMI category**						
Normal	1.00			1.00		
Underweight	0.45	0.17-1.24	0.123	0.41	0.13-1.22	0.108
Overweight	1.05	0.61-1.79	0.873	1.09	0.60-1.98	0.788
Obese	1.57	0.84-2.94	0.155	1.74	0.88-3.44	0.111
**Prolonged labor (>24 hours)**						
No	1.00			1.00		
Yes	2.45	1.47-4.11	0.001	2.46	1.37-4.40	0.003**
**Hypertension**						
No	1.00			1.00		
Yes	1.91	1.00-3.65	0.049	1.69	0.81-3.53	0.158
**Chronic cough**						
No	1.00			1.00		
Yes	1.70	0.84-3.44	0.143	1.75	0.79-3.84	0.166
**Chronic constipation**						
No	1.00			1.00		
Yes	1.73	0.95-3.14	0.073	1.53	0.78-2.98	0.216
**Neurological illness**						
No	1.00			1.00		
Yes	3.88	1.20-12.51	0.023	3.50	0.89-13.82	0.074

Crude odds ratios (COR); adjusted odds ratios (AOR); confidence intervals (CI); **p < 0.05 statistically significant

## Discussion

### Prevalence of urinary incontinence

This study found that 27.5% of women attending gynecologic outpatient clinics had UI, indicating that approximately one in four women in this referral-level outpatient population were affected. Prevalence was comparable between Kayunga (30.3%) and Jinja (25.4%), stress UI was the predominant subtype, and increasing age, no formal education, high parity, and prolonged labor were the main independent correlates.

The observed prevalence aligns with estimates from other healthcare-contact populations assessed using symptom-based instruments. It is higher than the 12% reported in a tertiary gynecology clinic in Ghana, but it is comparable to clinic and healthcare-contact estimates reported elsewhere in Africa and outside Africa [9,16,17].

The lower prevalence reported in the Ghanaian study may reflect contextual and measurement differences rather than true epidemiological divergence. In the present study, UI was identified using the interviewer-administered Questionnaire for Urinary Incontinence Diagnosis rather than urodynamic confirmation. Symptom-based tools remain appropriate for epidemiologic studies, but disclosure and reporting can still be shaped by stigma, privacy, and cultural interpretation of leakage symptoms.

Higher clinic-based prevalence estimates reported in other outpatient populations, including 44% among Omani women and higher burdens among peri- and postmenopausal women in Nepal, likely reflect older age structures and clinic populations enriched with women at higher baseline risk [18,19]. In contrast, the median age in the current study was 32 years, which may partly explain the lower prevalence observed here.

Variation in reported prevalence across studies is also influenced by measurement heterogeneity. Differences in symptom-based instruments, recall periods, interviewer versus self-administration, and case definitions can produce important variation even when populations are broadly similar [4,5].

Despite the clinic-based setting, underreporting likely remains important. Many women may normalize urinary leakage after childbirth or with aging, feel embarrassed to disclose symptoms, or regard UI as a low priority compared with the complaint that prompted the clinic visit [17,20]. Limited pelvic floor health services and weak routine screening within resource-constrained health systems may further contribute to underdiagnosis.

### Clinical types of urinary incontinence

Among women with UI, stress UI was the most common clinical type, followed by urgency UI and mixed UI. This pattern is clinically plausible in a population with high exposure to vaginal delivery, multiparity, and prolonged labor, all of which may contribute to pelvic floor muscle, fascial, and urethral support injury.

The predominance of stress UI is consistent with evidence from East Africa and other regions. Ugandan data from women with severe pelvic floor compromise after obstetric fistula repair and population-based data from rural Tanzania similarly highlight a strong stress component [11,12]. Repeated pregnancy-related loading of the pelvic floor and childbirth-related tissue injury provide a biologically plausible explanation for this pattern.

International comparisons further contextualize the findings. Clinic-based studies from Nepal and Oman also reported stress UI as the predominant subtype, whereas some primary care samples report relatively higher urgency or mixed symptom burdens, likely reflecting differences in age structure, comorbidity, and care-seeking pathways [18,19].

At the population level, large surveys such as the National Health and Nutrition Examination Survey (NHANES) report a similar ordering of subtypes, with stress UI as the most common form, followed by urgency UI and mixed UI [5,7].

The subtype classification in this study was based on QUID thresholds derived from symptom responses obtained in private interviews. Although this approach is suitable for outpatient epidemiologic research, symptom-based classification does not replace clinical examination or urodynamic assessment, and some misclassification remains possible [15].

Overall, the predominance of stress UI in this study strengthens the case for routine screening, early pelvic floor assessment, and postpartum counseling for women with repeated childbirth exposure or prolonged labor histories.

### Factors associated with urinary incontinence

In this study, UI was independently associated with increasing age, lower educational attainment, parity of four or more deliveries, and a history of prolonged labor, whereas other reproductive, clinical, and lifestyle variables did not retain independent associations after adjustment. For BMI, hypertension, chronic cough, chronic constipation, and neurological illness, the adjusted estimates should be interpreted cautiously because some were imprecise and the study may have had limited power for less frequent clinical exposures. These findings support the view that life course exposure and obstetric pelvic floor injury remain important correlates of UI in this setting.

Within the Ugandan context, these findings are concordant with clinic-based and obstetric evidence linking age-related vulnerability and childbirth-related pelvic floor trauma with urinary incontinence [9,12]. Advancing age may reflect reduced connective tissue resilience and neuromuscular support, while repeated childbirth and prolonged labor may compound pelvic floor strain over time.

Comparable patterns have been reported in neighboring East African settings, where older age and adverse obstetric histories, particularly prolonged or difficult labor, show strong associations with urinary incontinence [10,11].

Across other African gynecology clinic populations, age and socioeconomic indicators, including education, have also shown associations with UI. Lower education may influence symptom recognition, health literacy, care seeking, and opportunities for preventive counseling or timely obstetric care [9].

Evidence from non-African low- and middle-income settings similarly identifies increasing age, lower education, and obstetric trauma as important correlates of UI [18,19,21]. The association with prolonged labor in the present study is especially important because it points to a potentially modifiable health system factor through improved intrapartum monitoring and timely obstetric intervention.

Globally, population-based studies consistently demonstrate strong age gradients and life course effects of childbirth-related pelvic floor injury [3,5,22]. The current findings therefore fit well within the broader epidemiologic literature while adding referral-clinic evidence from Uganda.

Taken together, these results emphasize that routine outpatient screening should particularly target older women, women with limited formal education, women with high parity, and women reporting previous prolonged labor.

These findings support practical integration of UI screening into routine gynecologic outpatient care in similar referral settings. A brief symptom screen such as the QUID could be administered during triage or nursing assessment, with referral pathways for counseling, pelvic floor muscle training, and specialist review when indicated. Integration with maternal, postpartum, and reproductive health services may also help identify women with persistent or cumulative pelvic floor symptoms who do not volunteer leakage unless asked. In resource-limited clinics, task-sharing with trained nurses, midwives, or other non-specialist providers may improve early identification and counseling while reserving specialist care for complex or refractory cases.

### Strengths and limitations

This study had several strengths. It included women from two regional referral hospitals, used a validated symptom-based tool for classifying UI, and evaluated a broad range of sociodemographic, obstetric, and clinical factors within routine gynecologic outpatient practice.

Several limitations should be considered. The cross-sectional design precludes causal inference, and the associations should not be interpreted as temporal or causal relationships. Consecutive sampling in referral hospitals may have introduced selection bias and limits generalizability to community populations and lower-level facilities. Some covariates, including comorbidities and past obstetric experiences, relied partly on self-report and may have been affected by recall error or incomplete prior documentation. UI was classified using a symptom-based QUID screen rather than clinical examination or urodynamic confirmation, so some misclassification is possible. Residual confounding also remains possible because some potentially relevant variables, including menopausal status and detailed pelvic floor treatment history, were not measured. Some adjusted estimates, particularly for the oldest age group and less frequent clinical exposures, had wide confidence intervals, indicating limited precision; these results should therefore be interpreted as suggestive rather than definitive. In addition, women older than 55 years, pregnant women, and women in the immediate postpartum period were excluded, limiting generalizability to those groups.

## Conclusion

Urinary incontinence is common among women attending gynecologic outpatient clinics in Uganda, affecting approximately one in four attendees. Stress UI is the predominant subtype. Increasing age, no formal education, high parity, and a history of prolonged labor were independently associated with UI.

These findings support routine UI screening in gynecologic outpatient services, strengthened intrapartum care to address prolonged labor, postpartum pelvic floor counseling for high-risk women, and health system efforts to improve early recognition and care-seeking.

## Supporting information

S1 Dataset De-identified participant-level dataset and data dictionary underlying the findings. (XLS)

S1 Appendix Questionnaire for Urinary Incontinence Diagnosis (QUID) scoring guide. (DOCX)

S1 Checklist STROBE checklist for cross-sectional studies. The STROBE Statement checklist is reproduced under the terms of the Creative Commons Attribution 4.0 International (CC BY 4.0) License. See the STROBE Initiative for further information.
(DOCX)

## References

[1] Abrams P, Cardozo L, Fall M, Griffiths D, Rosier P, Ulmsten U, et al. The standardisation of terminology of lower urinary tract function: report from the Standardisation Sub-committee of the International Continence Society. Neurourol Urodyn. 2002;21(2):167–78. doi: 10.1002/nau.10052 11857671

[2] Hunskaar S, Lose G, Sykes D, Voss S. The prevalence of urinary incontinence in women in four European countries. BJU Int. 2004;93(3):324–30. doi: 10.1111/j.1464-410x.2003.04609.x 14764130

[3] Minassian VA, Hagan KA, Erekson E, Austin AM, Carmichael D, Bynum JPW, et al. The natural history of urinary incontinence subtypes in the Nurses’ Health Studies. Am J Obstet Gynecol. 2020;222(2):163.e1-163.e8. doi: 10.1016/j.ajog.2019.08.023 31449803 PMC6995407

[4] Bedretdinova D, Fritel X, Panjo H, Ringa V. Prevalence of Female Urinary Incontinence in the General Population According to Different Definitions and Study Designs. Eur Urol. 2016;69(2):256–64. doi: 10.1016/j.eururo.2015.07.043 26259998

[5] Abufaraj M, Xu T, Cao C, Siyam A, Isleem U, Massad A. Prevalence and trends in urinary incontinence among women in the United States, 2005–2018. Am J Obstet Gynecol. 2021;225(2):166.e1-166.e12. doi: 10.1016/j.ajog.2021.03.016 33727114

[6] Peyrat L, Haillot O, Bruyere F, Boutin JM, Bertrand P, Lanson Y. Prevalence and risk factors of urinary incontinence in young and middle-aged women. BJU Int. 2002;89(1):61–6. doi: 10.1046/j.1464-4096.2001.01813.x 11849162

[7] Lee UJ, Feinstein L, Ward JB, Kirkali Z, Martinez-Miller EE, Matlaga BR, et al. Prevalence of Urinary Incontinence among a Nationally Representative Sample of Women, 2005-2016: Findings from the Urologic Diseases in America Project. J Urol. 2021;205(6):1718–24. doi: 10.1097/JU.0000000000001634 33605795

[8] Quaghebeur J, Bush M, Shkarupa D, Wyndaele J-J, De Wachter S. A brief physiology and pathophysiology of the bladder. Ann Transl Med. 2024;12(2):24. doi: 10.21037/atm-23-1770 38721465 PMC11075957

[9] Ofori AA, Osarfo J, Agbeno EK, Azanu WK, Opare-Addo HS. Prevalence and determinants of non-fistulous urinary incontinence among Ghanaian women seeking gynaecologic care at a teaching hospital. PLoS One. 2020;15(8):e0237518. doi: 10.1371/journal.pone.0237518 32810136 PMC7433879

[10] Maroyi R, Mwambali N, Moureau MK, Keyser LE, McKinney JL, Brown HW, et al. Prevalence of urinary incontinence in pregnant and postpartum women in the Democratic Republic of Congo. Int Urogynecol J. 2021;32(7):1883–8. doi: 10.1007/s00192-021-04885-w 34152428

[11] Masenga GG, Shayo BC, Msuya S, Rasch V. Urinary incontinence and its relation to delivery circumstances: A population-based study from rural Kilimanjaro, Tanzania. PLoS One. 2019;14(1):e0208733. doi: 10.1371/journal.pone.0208733 30673696 PMC6343883

[12] Nardos R, Jacobson L, Garg B, Wall LL, Emasu A, Ruder B. Characterizing persistent urinary incontinence after successful fistula closure: the Uganda experience. Am J Obstet Gynecol. 2022;227(1):70.e1-70.e9. doi: 10.1016/j.ajog.2022.03.008 35283092

[13] Nzinga Luzolo A-M, Dilu Mabiala E, Bilo Mbaki I, Ngereza Kibimbi P, Bope Matshinga N, Kasonga R-S. Epidemiological Profile and Attitudes of Pregnant Women Toward Urinary Incontinence: A Single-Center Cross-Sectional Study. Int Urogynecol J. 2024;35(3):521–6. doi: 10.1007/s00192-023-05718-8 38189851

[14] Whiting D, Shane AI, Pope R, Payne S, Venn S. Female urinary incontinence in sub-Saharan Africa. BJU Int. 2022;130(5):543–9. doi: 10.1111/bju.15903 36161452

[15] Bradley CS, Rovner ES, Morgan MA, Berlin M, Novi JM, Shea JA, et al. A new questionnaire for urinary incontinence diagnosis in women: development and testing. Am J Obstet Gynecol. 2005;192(1):66–73. doi: 10.1016/j.ajog.2004.07.037 15672005

[16] Junqueira JB, Santos VLC de G. Urinary incontinence in hospital patients: prevalence and associated factors. Rev Lat Am Enfermagem. 2018;25:e2970. doi: 10.1590/1518-8345.2139.2970 29319744 PMC5768210

[17] Lasserre A, Pelat C, Guéroult V, Hanslik T, Chartier-Kastler E, Blanchon T, et al. Urinary incontinence in French women: prevalence, risk factors, and impact on quality of life. Eur Urol. 2009;56(1):177–83. doi: 10.1016/j.eururo.2009.04.006 19376639

[18] Adhikari A, Poudel A. Urinary incontinence among peri and postmenopausal women. JCMC. 2023;13(3):79–82. doi: 10.54530/jcmc.1383

[19] Seshan V, Francis F, Raghavan D, Arulappan J, Hashmi IA, Prince EJ, et al. Prevalence of Urinary Incontinence and its Relationship With Sociodemographic and Obstetrical Variables Among Omani Women. SAGE Open Nurs. 2023;9:23779608231173803. doi: 10.1177/23779608231173803 37223218 PMC10201158

[20] Przydacz M, Chlosta M, Chlosta P. Population-Level Prevalence, Bother, and Treatment Behavior for Urinary Incontinence in an Eastern European Country: Findings from the LUTS POLAND Study. J Clin Med. 2021;10(11):2314. doi: 10.3390/jcm10112314 34073165 PMC8199423

[21] Abduldaiem AO, Al Issa H, Selim M, Kofi M. Prevalence and risk factors of urinary incontinence among adult Saudi women in Riyadh, Saudi Arabia. Int J Med Health Res. 2020;6(7):56–64.

[22] Du Y, Wang P, Chen Y, Liu Q, Wang L, Jin H, et al. Fetal birthweight and maternal urinary incontinence in Chinese primiparas: a population-based study. BMC Public Health. 2025;25(1):754. doi: 10.1186/s12889-025-21849-7 39994577 PMC11853197

