# Supplementary material for: Urinary incontinence in Ugandan gynecologic outpatient clinics: Prevalence, clinical types, and associated factors
Source: PLOS Glob Public Health. 2026 Sep 15;6(9):e0007221. doi: 10.1371/journal.pgph.0007221 (PMC13577378; doi:10.1371/journal.pgph.0007221)
Supplement: Supplementary file 1 [file pgph.0007221.s002.docx]

**Study variables and Questionnaire for Urinary Incontinence Diagnosis (QUID) scoring guide**

*Urinary Incontinence in Ugandan Gynecologic Outpatient Clinics: Prevalence, Clinical Types, and Associated Factors*

**Section A. Sociodemographic and behavioral characteristics**

| **Variable** | **Response / definition** |
| --- | --- |
| Age | Completed years; analytic groups: <30, 30-39, 40-49, 50+ years |
| Marital status | Married / Not-married |
| Place of residence | Rural / Urban |
| Level of education | No formal education / Secondary / Primary / Tertiary |
| Occupation | Formal employment / Unemployed / Non formal employment |
| Monthly household income | <200,000 UGX / 200,000-400,000 UGX / >400,000 UGX |
| History of smoking | No / Yes |
| Regular physical activity | No / Yes; regular physical activity defined as >=3 days/week |
| Ever use of contraception | No / Yes |

**Section B. Reproductive characteristics**

| **Variable** | **Response / definition** |
| --- | --- |
| Parity | Number of prior births at 28 weeks of gestation or more; analytic groups: 0 / 1 / 2 / 3 / 4+ |
| History of macrosomic baby | No / Yes; macrosomia defined as birthweight >4 kg |
| Mode of last delivery | Vaginal / Cesarean |
| Place of last delivery | Health facility / Home |
| History of prolonged labor | No / Yes; any previous labor reported to have lasted >24 hours |

**Section C. Clinical and anthropometric characteristics**

| **Variable** | **Response / definition** |
| --- | --- |
| Height | Measured or recorded height used with weight to calculate body mass index |
| Weight | Measured or recorded weight used with height to calculate body mass index |
| Body mass index | kg/m²; categorized as Underweight / Normal / Overweight / Obese using standard thresholds |
| Hypertension | No / Yes; self-report of a prior clinician diagnosis |
| Diabetes mellitus | No / Yes; self-report of a prior clinician diagnosis |
| Chronic cough | No / Yes |
| Chronic constipation | No / Yes |
| Neurological illness | No / Yes |
| History of abdominal, pelvic, or spine surgery | No / Yes |

**Section D. Questionnaire for Urinary Incontinence Diagnosis (QUID)**

Participants were asked how often they experienced each urinary symptom. Items 1-3 form the stress subscale and items 4-6 form the urgency subscale.

| **Item** | **Question** | **Domain** |
| --- | --- | --- |
| 1 | Do you leak urine when you cough or sneeze? | Stress |
| 2 | Do you leak urine when bending or lifting something? | Stress |
| 3 | Do you leak urine when walking quickly, jogging, or exercising? | Stress |
| 4 | Do you leak urine while undressing to urinate? | Urgency |
| 5 | Do you leak before reaching the toilet due to a strong urge? | Urgency |
| 6 | Do you rush to the bathroom due to a sudden, strong need to urinate? | Urgency |

**Response scoring**

| **None** | **Rarely** | **Once in a while** | **Often** | **Most of the time** | **All the time** |
| --- | --- | --- | --- | --- | --- |
| 0 | 1 | 2 | 3 | 4 | 5 |

**Score calculation and classification**

**•** Stress subscale score: sum of items 1-3 (possible range 0-15).

**•** Urgency subscale score: sum of items 4-6 (possible range 0-15).

**•** No urinary incontinence: stress score <4 and urgency score <6.

**•** Stress urinary incontinence: stress score >=4 and urgency score <6.

**•** Urgency urinary incontinence: urgency score >=6 and stress score <4.

**•** Mixed urinary incontinence: stress score >=4 and urgency score >=6.

Reference: Bradley CS, Rovner ES, Morgan MA, Berlin M, Novi JM, Shea JA, et al. A new questionnaire for urinary incontinence diagnosis in women: development and testing. Am J Obstet Gynecol. 2005;192(1):66-73. <https://doi.org/10.1016/j.ajog.2004.07.037>
