# Supplementary material for: Urinary incontinence in Ugandan gynecologic outpatient clinics: Prevalence, clinical types, and associated factors
Source: PLOS Glob Public Health. 2026 Sep 15;6(9):e0007221. doi: 10.1371/journal.pgph.0007221 (PMC13577378; doi:10.1371/journal.pgph.0007221)
Supplement: Supplementary file 2 [file pgph.0007221.s003.docx]

**STROBE checklist for cross-sectional studies**

*Urinary Incontinence in Ugandan Gynecologic Outpatient Clinics: Prevalence, Clinical Types, and Associated Factors*

| **Section** | **Item** | **STROBE reporting item (abbreviated)** | **Where reported in this manuscript** |
| --- | --- | --- | --- |
| **Title and abstract** | 1 | State the cross-sectional design and give a balanced abstract. | Reported - Abstract, pp. 2-3. The Methods sentence identifies a cross-sectional study; the abstract summarizes methods, prevalence, subtypes, associated factors, and conclusions. |
| **Introduction** | 2 | Explain the scientific background and rationale. | Reported - Introduction, pp. 3-5. |
| **Introduction** | 3 | State the study objectives and any prespecified hypotheses. | Reported - Introduction, pp. 4-5. Objectives are stated; no prespecified hypothesis is reported. |
| **Methods** | 4 | Present the study design early. | Reported - Study design and setting, p. 5. |
| **Methods** | 5 | Describe setting, study sites, and relevant dates. | Reported - Study design and setting, p. 5 (Kayunga and Jinja Regional Referral Hospitals; 10 July-8 October 2025). |
| **Methods** | 6 | Give eligibility criteria and participant-selection methods. | Reported - Study population and eligibility criteria, pp. 5-6; Sample size and sampling, p. 6. |
| **Methods** | 7 | Define outcomes, predictors/covariates, and diagnostic criteria. | Reported - Variables, p. 7; QUID thresholds define UI and its clinical types. |
| **Methods** | 8 | Describe data sources and measurement methods. | Reported - Study procedures and data collection, pp. 6-7; Variables, p. 7; S1 Appendix provides the QUID scoring guide and variable definitions. |
| **Methods** | 9 | Describe measures used to address potential bias. | Reported across sections - Methods, pp. 6-8 (consecutive recruitment, trained interviewers, private administration, validated QUID); Strengths and limitations, p. 20 discusses selection, recall, misclassification, and residual confounding. |
| **Methods** | 10 | Explain how the study size was determined. | Reported - Sample size and sampling, p. 6 (Kish and Leslie formula; target n=374 after 10% allowance). |
| **Methods** | 11 | Explain handling and categorization of quantitative variables. | Reported - Variables and Statistical analysis, pp. 7-8; category boundaries are shown in Tables 1-3 and 5-6. |
| **Methods** | 12 | Describe statistical methods, confounding control, missing-data handling, and other analyses as applicable. | Reported - Statistical analysis, pp. 7-8. Bivariate and multivariable logistic regression, p<0.20 screening, 95% CIs, diagnostics, and complete-case analysis are described. No interaction or sensitivity analyses are reported. |
| **Results** | 13 | Report participant numbers, non-participation, and a flow diagram. | Partly reported - Participant flow, p. 9 and Fig 1: 419 assessed, 374 enrolled and analyzed, 45 excluded or not enrolled. Specific reasons for the 45 are not itemized. |
| **Results** | 14 | Describe participant characteristics and missing data. | Reported - Tables 1-3, pp. 9-11. All enrolled participants were analyzed; complete-case analysis is stated on p. 8. |
| **Results** | 15 | Report outcome-event counts or summary measures. | Reported - Prevalence and UI types, pp. 11-12; 103/374 had UI, with subtype distribution reported. |
| **Results** | 16 | Report crude/adjusted estimates, precision, category boundaries, and absolute risk when relevant. | Reported - Tables 5-6, pp. 13-15, with crude and adjusted ORs, 95% CIs, p values, and category boundaries. Absolute-risk translation is not applicable to these cross-sectional odds-ratio analyses. |
| **Results** | 17 | Report other analyses performed. | Reported - Site-specific prevalence and bivariate analyses, pp. 12-15. No additional subgroup, interaction, or sensitivity analyses are reported. |
| **Discussion** | 18 | Summarize the key results in relation to the objectives. | Reported - Discussion, pp. 15-20; Conclusion, pp. 20-21. |
| **Discussion** | 19 | Discuss limitations, potential bias, and imprecision. | Reported - Strengths and limitations, p. 20, including cross-sectional design, referral sampling, self-report, QUID misclassification, residual confounding, and wide CIs. |
| **Discussion** | 20 | Interpret findings cautiously in light of limitations and other evidence. | Reported - Discussion, pp. 15-20. |
| **Discussion** | 21 | Discuss generalizability of the findings. | Reported - Strengths and limitations, p. 20; Conclusion, pp. 20-21. Generalizability is limited to similar referral-level settings and excluded groups are identified. |
| **Other information** | 22 | State funding and the funder's role. | Reported in PLOS submission metadata - the authors received no specific funding for this work; funder role is therefore not applicable. |
