# Supplementary material for: Urinary incontinence in Ugandan gynecologic outpatient clinics: Prevalence, clinical types, and associated factors
Source: PLOS Glob Public Health. 2026 Sep 15;6(9):e0007221. doi: 10.1371/journal.pgph.0007221 (PMC13577378; doi:10.1371/journal.pgph.0007221)
Supplement: Supplementary file 6 [file pgph.0007221.s007.docx]

**Response to Reviewers**

Urinary Incontinence in Ugandan Gynecologic Outpatient Clinics: Prevalence, Clinical Types, and Associated Factors

PLOS Global Public Health | PGPH-D-25-04011R1

Dear Professor Gyasi and Reviewers,

We thank the Editor and reviewers for their careful reassessment of the manuscript. We have revised the manuscript to address each remaining point, with changes made in the Introduction, Methods, Results, Discussion, Conclusion, and References as described below.

# Editor

**Comment E.1:**

Please address the remaining reviewer comments and respond appropriately.

**Response:**

We addressed each remaining reviewer comment. The corresponding revisions are summarized below.

# Reviewer #1

**Comment R1.1:**

All comments have been addressed; meet the requirements as much as possible.

**Response:**

We thank Reviewer #1 for confirming that the prior comments were addressed. No additional manuscript changes were requested.

# Reviewer #3

**Comment R3.1:**

Clarify the novelty of the study compared with existing literature in sub-Saharan Africa.

**Response:**

We revised the final paragraph of the Introduction to specify that the study adds referral-level Ugandan evidence from routine gynecologic outpatient clinics using QUID-based assessment of prevalence, clinical subtypes, and associated factors among non-pregnant women. This appears in the Introduction.

**Comment R3.2:**

Expand the limitations regarding selection bias, cross-sectional design, self-reported variables, and symptom-based classification.

**Response:**

We expanded the Strengths and limitations section to address the cross-sectional design, consecutive sampling in referral hospitals, potential selection bias, limited generalizability, self-reported covariates, and possible misclassification from symptom-based QUID screening.

**Comment R3.3:**

Acknowledge potential confounders such as menopausal status and interpret BMI and other clinical variables more critically.

**Response:**

We added residual confounding to the limitations, including unmeasured menopausal status and detailed pelvic floor treatment history. We also revised the Discussion to interpret BMI and other clinical variables cautiously because some adjusted estimates were imprecise and less frequent exposures may have been underpowered.

**Comment R3.4:**

Briefly mention model diagnostics, wide confidence intervals, and residual confounding.

**Response:**

We revised the Statistical analysis section to state that model diagnostics included assessment of multicollinearity using variance inflation factors and review of model classification performance. We also added discussion of wide confidence intervals and residual confounding in the limitations.

**Comment R3.5:**

Elaborate on public health implications, including routine screening, integration into maternal/reproductive health services, and task-sharing.

**Response:**

We added a Discussion paragraph on practical implementation. The revision addresses QUID screening during triage or nursing assessment, referral pathways, integration with maternal, postpartum, and reproductive health services, and task-sharing with trained non-specialist providers.

**Comment R3.6:**

Avoid causal language given the cross-sectional design.

**Response:**

We reviewed the manuscript for causal wording and revised statements to use associative language where appropriate. The Discussion and Conclusion now describe findings as associations or correlates rather than causal relationships.

**Comment R3.7:**

Some tables are dense and could be simplified or moved to supplementary materials.

**Response:**

We considered this suggestion carefully. Because the tables present participant characteristics, subgroup prevalence, and regression outputs that support the main objectives, we retained them in the main manuscript for transparency and reviewed the labels for clarity.

**Comment R3.8:**

Minor grammatical and stylistic edits are needed throughout the manuscript.

**Response:**

We made targeted grammatical and stylistic edits in revised passages and corrected mechanical issues, including hyphenation and spacing, while avoiding broad rewriting outside the requested revisions.

**Comment R3.9:**

Reduce redundant phrasing in the Introduction and Discussion.

**Response:**

We streamlined repeated wording in the Introduction and revised the Discussion so that the added public health implications complement rather than duplicate the Conclusion.

# Reviewer #4

**Comment R4.1:**

The title is appropriate; the abstract is appropriate and corrected.

**Response:**

We thank Reviewer #4 for confirming that the title and abstract are appropriate. No further changes were requested for these sections.

**Comment R4.2:**

Introduction line 85: state Kayunga and Jinja Regional Referral Hospitals (RRH).

**Response:**

We revised the Introduction to state “Kayunga and Jinja Regional Referral Hospitals (RRH)” at the relevant first mention.

**Comment R4.3:**

Methods lines 92 and 98: state Kayunga and Jinja Regional Referral Hospitals (RRH).

**Response:**

We revised the Study design and setting and Study population and eligibility criteria sections to use “Kayunga and Jinja Regional Referral Hospitals (RRH)” as requested.

**Comment R4.4:**

Methods line 137 and Results line 171: state Body mass index (BMI).

**Response:**

We revised the Variables section and Results description of participant characteristics to define “body mass index (BMI)” at the requested locations.

**Comment R4.5:**

References: mention the DOI of the article with volume and issue number where applicable.

**Response:**

We reviewed and updated the reference list to include volume, issue, page or article number, and DOI information where available. No new references were added.
