# Supplementary material for: Urinary incontinence in Ugandan gynecologic outpatient clinics: Prevalence, clinical types, and associated factors
Source: PLOS Glob Public Health. 2026 Sep 15;6(9):e0007221. doi: 10.1371/journal.pgph.0007221 (PMC13577378; doi:10.1371/journal.pgph.0007221)
Supplement: Supplementary file 7 [file pgph.0007221.s004.docx]

PLOS Global Public Health

Review of the article titled “Prevalence, clinical types, and associated factors of urinary incontinence among women attending gynecologic outpatient clinics in Uganda: a cross-sectional study”

1. Title.

The title is appropriate. But the title should be written in title case.

2. Abstract.

Objective: Line 26: State ‘To determine’ instead of Determining
Methods: Line-29: State Kayunga and Jinja Regional Referral Hospitals (RRH)
Result: Line-35: State ‘stress UI’ instead of stress IU
Conclusion: Line-43: State ‘stress UI’ instead of stress urinary incontinence.

3. Introduction section.

Rationale for conducting the study is clear.
Line-87: State pelvic floor health (PFH).
Line 20 & 21: state 'can be' instead of 'will be'.

4. The Methods are described in sufficient details and the ethical concerns has been well described.
Line-99: State ‘from 10^th^ July to 8^th^ October, 2025’ instead of ‘between 10^th^ July and 8^th^ October 2025’
Line-111: Nardos et al., is not required as reference, Vancouver referencing style is enough.
Line 134: Define prolong labor in case of multipara and primipara.

5. Results section. 
Line-166: Median age 32 years (interquartile range: 26-39) had not shown in Table 1.

Line-173: Add (N=374) with title of Table 2

Line-175: Add (N=374) with title of Table 3

Line-179: State ‘near similar’ instead of similar

Line-183: State ‘UI’ instead of urinary incontinence and stress UI instead of stress urinary incontinence

Line 184: State ‘Mixed UI’ instead of mixed urinary incontinence

Line 185: State ‘urgency UI’ instead of urgency urinary incontinence

6. Discussion section

Line-226: Write the elaboration of ICIQ-SF

Line-251: State ‘UI’ instead of urinary incontinence and stress UI instead of stress urinary incontinence

Line 252: State ‘urge and mixed UI’ instead of urge and mixed urinary incontinence

Line 256: State ‘stress UI’ instead of stress urinary incontinence

Line 259: State ‘stress UI’ instead of stress urinary incontinence

Line 267: Define NHANES

7.  References.

Line-386: Mention the name of the journal in abbreviated form

Line-392: Mention the name of the journal in abbreviated form

Line-395: Mention the name of the journal in abbreviated form

Line-399: Mention the name of the journal in abbreviated form

Line-409: Mention the name of the journal in abbreviated form with volume and issue number

Line-411: Mention the name of the journal in abbreviated form

Line-424: Write the name of the article in Title case and Journal name in correct abbreviated form

Line-427: Mention the name of the journal with volume and issue number.

Line-434: Mention the name of journal in abbreviated form

8. Standard of English for publication.

The English is clear to understand with some **incomprehensible parts.** The article requires thorough proof-reading before publishing.
