# Supplementary material for: Urinary incontinence in Ugandan gynecologic outpatient clinics: Prevalence, clinical types, and associated factors
Source: PLOS Glob Public Health. 2026 Sep 15;6(9):e0007221. doi: 10.1371/journal.pgph.0007221 (PMC13577378; doi:10.1371/journal.pgph.0007221)
Supplement: Supplementary file 8 [file pgph.0007221.s005.docx]

**Response to Reviewers**

Manuscript ID: PGPH-D-25-04011

Title: Urinary Incontinence in Ugandan Gynecologic Outpatient Clinics: Prevalence, Clinical Types, and Associated Factors

Dear Editor and Reviewers,

We sincerely thank the editor and all reviewers for their careful and constructive assessment of our manuscript. We have revised the manuscript extensively to improve clarity, methodological transparency, internal consistency, figure quality, discussion focus, and reporting style.

Major revisions include a more concise title, a revised abstract, expanded introduction and methods sections, standardized terminology throughout, correction of the urinary incontinence subtype distribution, redrawn figures, clearer table titles and denominators, explicit strengths and limitations, a revised conclusion, and a refined reference list.

Below, we provide a point by point response to each comment. Reviewer comments are reproduced in italics, followed by our responses and a summary of the corresponding manuscript changes.

# Editor and Journal Requirements

**Comment:** *The manuscript requires meticulous revision, and separate high resolution figure files should be provided at resubmission.*

**Response:** The manuscript has undergone a comprehensive revision addressing all methodological, interpretive, and reporting concerns raised by the reviewers. All figures have been redrawn to improve clarity, formatting, and resolution.

In accordance with journal requirements, separate main figure files in .tif or .eps format have been prepared for submission. The figures in the manuscript were redrawn for clarity and resolution, and the final figure files should be uploaded separately with the revised submission.

# Reviewer 1

**Comment:** *Title may be more concise.*

**Response:** The title has been shortened and converted to title case. The revised title is: “Urinary Incontinence in Ugandan Gynecologic Outpatient Clinics: Prevalence, Clinical Types, and Associated Factors.”

**Comment:** *In the introduction, add details about sociocultural factors affecting UI diagnosis and treatment, explain healthcare system barriers, and reduce unnecessary citation density.*

**Response:** The introduction has been revised to include sociocultural and health system explanations for underrecognition of UI, including stigma, normalization of leakage after childbirth or with aging, limited pelvic floor health services, and competing priorities in resource constrained systems. Redundant wording was removed and the section was streamlined.

**Comment:** *In the methods, clarify the sampling technique and whether pregnant women and women with recent childbirth were included or excluded.*

**Response:** The Methods section now explicitly states that consecutive sampling was used. Eligibility criteria were revised to clarify that the study included non-pregnant women aged 18 to 55 years attending gynecologic outpatient clinics, and excluded women who were currently pregnant or within six weeks postpartum.

**Comment:** *In Table 4, prevalence of UI could be compared across different subgroups.*

**Response:** To address this point, the results narrative now explicitly states that subgroup specific UI prevalence is presented in the bivariate table, and the title of the revised bivariate table was changed to “Subgroup Prevalence and Bivariate Logistic Regression of Factors Associated with UI (N = 374).” This makes it clearer that subgroup comparisons are shown alongside the crude analysis.

**Comment:** *In the discussion, explain the sociocultural factors more elaborately.*

**Response:** The Discussion now includes a fuller interpretation of stigma, symptom normalization, delayed disclosure, limited routine screening, and restricted access to pelvic floor health services as likely contributors to underreporting and underdiagnosis.

# Reviewer 2

**Comment:** *Explain the abbreviation PFH and briefly explain the different types of urinary incontinence and their unique causes in the introduction.*

**Response:** The abbreviation PFH is now defined as pelvic floor health at first mention. The Introduction also now briefly explains stress UI, urgency UI, and mixed UI, and outlines their main clinical mechanisms.

**Comment:** *Why were these clinics chosen?*

**Response:** The Study design and setting subsection now explains that Kayunga Regional Referral Hospital and Jinja Regional Referral Hospital are public regional referral facilities providing specialist obstetric and gynecologic services to predominantly rural and peri-urban populations in central and eastern Uganda.

**Comment:** *Explain the reason for choosing women between 18 and 55 years in the methodology.*

**Response:** The eligibility criteria now state that the age range was selected to focus on reproductive and early post-reproductive women commonly managed in gynecologic outpatient clinics while limiting confounding from frailty related incontinence at advanced ages.

**Comment:** *Kindly summarise the findings clearly in the first paragraph of the discussion.*

**Response:** The first paragraph of the Discussion has been rewritten to summarise the main findings clearly, including the overall prevalence, site comparison, predominant clinical subtype, and the key independent correlates.

**Comment:** *Clarify the statement that the consistency suggests a shared burden shaped by similar population risk profiles and care-seeking patterns.*

**Response:** This interpretation has been rewritten in simpler language and grounded directly in the study findings by explicitly stating that prevalence was comparable between Kayunga and Jinja, which suggests that the outpatient burden was not driven by one site alone.

**Comment:** *Simplify the explanation comparing this study with higher prevalence estimates elsewhere, and use figures where possible.*

**Response:** The comparative discussion was simplified. It now explains that higher outpatient estimates reported elsewhere, including about 44% in Oman and higher burdens among peri and postmenopausal women in Nepal, are likely influenced by older age structures and higher risk clinic populations. The revised text links these comparisons to the current study’s younger median age.

**Comment:** *There is no explanation of why stress urinary incontinence was the predominant type. The discussion also does not align well with the conclusions or recommendations.*

**Response:** The subtype discussion was rewritten to explain why stress UI predominated in this setting, emphasizing vaginal delivery, multiparity, and prolonged labor as plausible pathways to pelvic floor and urethral support injury. The final part of the manuscript was also restructured so that the conclusion and practice implications align directly with the presented findings.

# Reviewer 3

**Comment:** *The data availability statement did not meet PLOS standards.*

Response: The Data Availability statement was revised to state that the de-identified participant-level dataset and data dictionary underlying the findings are uploaded as a supporting information file and are provided as S1 Dataset.

**Comment:** *The reported subtype distribution was inconsistent between the abstract and the results section.*

**Response:** This inconsistency has been corrected throughout the manuscript. The final distribution is now reported consistently in the abstract, results, figure caption, and discussion as stress UI 44.7%, urgency UI 30.1%, and mixed UI 25.2%.

**Comment:** *The manuscript needs consistency in terminology, figure and table referencing, units, and denominators.*

**Response:** Terminology has been standardized throughout. The manuscript now consistently uses “gynecologic,” “stress UI,” “urgency UI,” and “mixed UI.” Figure references were standardized to “Fig,” table titles were numbered manually, and the denominators in the bivariate and multivariable table titles were corrected from N = 103 to N = 374.

# Reviewer 4

**Comment:** *The title should be written in title case.*

**Response:** This has been corrected.

**Comment:** *In the abstract, state “To determine” instead of “Determining,” use RRH after the hospital names, and standardize stress UI terminology.*

**Response:** The abstract was revised. The objective wording was integrated into the Background sentence, the hospital names were standardized to Kayunga and Jinja Regional Referral Hospitals (RRH), and all subtype terminology was standardized to stress UI, urgency UI, and mixed UI.

**Comment:** *In the introduction, define pelvic floor health (PFH), and replace “will be” with “can be” where appropriate.*

**Response:** PFH is now defined at first mention. The Introduction was also copyedited for grammar and wording consistency.

**Comment:** *In the methods, revise the study date format, remove author naming from the sample size sentence in favor of Vancouver style, and define prolonged labor in the context of parity.*

**Response:** The Methods section now states that data were collected “from 10th July to 8th October, 2025.” The sample size paragraph now cites the prior Ugandan study using Vancouver numbering only. History of prolonged labor is now explicitly defined as any previous labor reported to have lasted more than 24 hours, irrespective of parity, because prior partograph records were not consistently available.

**Comment:** *In the results, show the median age in relation to Table 1, add N = 374 to Tables 2 and 3, and standardize UI terminology.*

**Response:** The descriptive results now report the median age clearly, and a note below Table 1 states that the median age of the overall sample was 32 years with an interquartile range of 26 to 39. Tables 2 and 3 now include N = 374 in their titles. UI terminology has also been standardized throughout the Results section.

**Comment:** *In the discussion, define ICIQ-SF and NHANES and standardize subtype terms.*

**Response:** The erroneous reference to ICIQ-SF in the discussion was removed because the study used QUID rather than ICIQ-SF for case classification. NHANES is now written in full as the National Health and Nutrition Examination Survey at first mention. Subtype terminology has been standardized throughout.

**Comment:** *In the references, journal names should use standard abbreviations.*

**Response:** The reference list was revised to improve consistency with Vancouver style and to use abbreviated journal names where appropriate.

**Comment:** *The article requires thorough proof reading before publishing.*

**Response:** The manuscript has undergone full language editing and consistency checks.

# Reviewer 5

**Comment:** *The methodology and statistical analysis are satisfactory, but the figures are unclear, the reference style needs revision, and copy editing is required.*

**Response:** Both figures were redrawn for clarity and improved resolution. Figure captions were also revised for clearer interpretation. The reference list was cleaned for greater style consistency, and the manuscript was copyedited throughout to correct grammar, terminology, and internal inconsistencies.

# Reviewer 6

**Comment:** *The objective in the abstract need not be a separate heading and may appear as the final sentence under Background.*

**Response:** We followed this suggestion. The objective was integrated into the final sentence of the Background section of the abstract.

**Comment:** *Reorder parts of the introduction to improve flow.*

**Response:** The introduction was reorganized and streamlined so that the prevalence and conceptual background now progress more smoothly from the global context to Africa and Uganda.

**Comment:** *Further describe the two hospitals, including their service level and populations served.*

**Response:** The Study design and setting subsection now gives a clearer description of the two hospitals as public regional referral facilities serving predominantly rural and peri-urban communities in central and eastern Uganda.

**Comment:** *State clearly that all women who attended were approached.*

**Response:** This wording has been corrected in the sampling subsection.

**Comment:** *Reference the sample size formula used.*

**Response:** The sample size subsection now explicitly states the Kish and Leslie single population proportion formula, n = Z²p(1-p)/d².

**Comment:** *Provide the QUID guideline as supporting information.*

Response: The supporting information captions now list the QUID scoring guide as S1 Appendix. This file should accompany the revised submission.

**Comment:** *Clarify the reliability of self reported hypertension and diabetes rather than extracting from case notes.*

**Response:** The Variables subsection now explains that these measures were based on self-report of a prior clinician diagnosis because complete historical case records were not uniformly available in the outpatient setting. The revised manuscript also now interprets these variables cautiously and includes this as a limitation.

**Comment:** *Clarify the rationale for using p < 0.20 as the criterion for multivariable model entry.*

**Response:** The Statistical analysis subsection now states that variables with p < 0.20 at bivariate analysis were entered into the multivariable model to avoid premature exclusion of potentially important confounders.

**Comment:** *Move the participant recruitment description into the Methods section.*

**Response:** This was addressed by expanding the Study procedures and data collection subsection to describe screening, consent, questionnaire administration, and QUID screening during the outpatient encounter.

**Comment:** *Give the full meaning of NHANES.*

**Response:** This has been corrected in the Discussion.

**Comment:** *Write strengths and limitations explicitly, and avoid a separate recommendation heading under Discussion.*

**Response:** The final section of the manuscript was restructured. A dedicated “Strengths and limitations” subsection was added, the duplicated recommendation heading was removed, and the paper now ends with a concise conclusion followed by integrated practice and policy implications.

**Comment:** *Figure 1 is not clear.*

**Response:** Figure 1 was redrawn with a clean flow diagram layout and a clearer caption.

# Minor Global Corrections

**Comment:** *Standardized “gynecologic” throughout the manuscript and removed inconsistent alternation with “gynecological.”*

**Response:** Addressed in the revised manuscript.

**Comment:** *Corrected the study figure captions, manually numbered all tables and figures, and replaced placeholder caption text.*

**Response:** Addressed in the revised manuscript.

**Comment:** *Corrected the bivariate and multivariable table titles so that both now carry the correct denominator of N = 374.*

**Response:** Addressed in the revised manuscript.

**Comment:** *Added explicit strengths and limitations.*

**Response:** Addressed in the revised manuscript.

**Comment:** *Removed the duplicate “Recommendations” section and consolidated the closing message into a stronger conclusion and implications paragraph.*

**Response:** Addressed in the revised manuscript.

We hope that the revised manuscript has addressed all concerns satisfactorily and we are grateful for the opportunity to improve the paper.

Sincerely,

Musa Kasujja, on behalf of all authors
