# Supplementary material for: Urinary incontinence in Ugandan gynecologic outpatient clinics: Prevalence, clinical types, and associated factors
Source: PLOS Glob Public Health. 2026 Sep 15;6(9):e0007221. doi: 10.1371/journal.pgph.0007221 (PMC13577378; doi:10.1371/journal.pgph.0007221)
Supplement: Supplementary file 9 [file pgph.0007221.s006.docx]

PLOS Global Public Health

Rereview of the article titled “Urinary Incontinence in Ugandan Gynecologic Outpatient Clinics: Prevalence, Clinical Types, and Associated Factors”

1. Title.

The title is appropriate.

2. Abstract.

It is appropriate and corrected.

3. Introduction section.

Line-85: State Kayunga and Jinja Regional Referral Hospitals (RRH)

4. The Methods section
Line-92: State Kayunga and Jinja Regional Referral Hospitals (RRH)

Line-98: State Kayunga and Jinja Regional Referral Hospitals (RRH)

Line-137: State Body mass index (BMI). Because, BMI category is mentioned in Table 5 & 6

5. Results section. 
Line-171: State Body mass index (BMI)

6.  References.

Mention the DOI of the article with volume and issue number where applicable
