# Supplementary material for: Urinary incontinence in Ugandan gynecologic outpatient clinics: Prevalence, clinical types, and associated factors
Source: PLOS Glob Public Health. 2026 Sep 15;6(9):e0007221. doi: 10.1371/journal.pgph.0007221 (PMC13577378; doi:10.1371/journal.pgph.0007221)
Supplement: Peer review history

# Peer review: Decision Letter 0

**DOI:** 10.1371/journal.pgph.0007221.r001

**Authors:** Razak M Gyasi

10 Feb 2026

PGPH-D-25-04011

Prevalence, clinical types, and associated factors of urinary incontinence among women attending gynecologic outpatient clinics in Uganda: a cross-sectional study

PLOS Global Public Health

Dear Dr. Kasujja,

Thank you for submitting your manuscript to PLOS Global Public Health. After careful consideration, we feel that it has merit but does not fully meet PLOS Global Public Health’s publication criteria as it currently stands. Therefore, we invite you to submit a revised version of the manuscript that addresses the points raised during the review process.

Dear Authors,

I asdecided to invite as amny reviewers as possible to comment on your paper and they have diligently and quickly provided their perspectives on your manuscript. These comments are important to improve the quality of your paper.

The issues raised range from the problematization, methods, and discussion of the paper. I would like to offer you the opportunity for a meticulos revision of your paper based on these important comments.

I look forward for receiving your revised  manuscript for further assessment thereof.

Thank you.

Please submit your revised manuscript by Mar 27 2026 11:59PM. If you will need more time than this to complete your revisions, please reply to this message or contact the journal office at globalpubhealth@plos.org. When you're ready to submit your revision, log on to https://www.editorialmanager.com/pgph/ and select the 'Submissions Needing Revision' folder to locate your manuscript file.

Please include the following items when submitting your revised manuscript:

- A letter that responds to each point raised by the editor and reviewer(s). You should upload this letter as a separate file labeled 'Response to Reviewers'.
- A marked-up copy of your manuscript that highlights changes made to the original version. You should upload this as a separate file labeled 'Revised Manuscript with Track Changes'.
- An unmarked version of your revised paper without tracked changes. You should upload this as a separate file labeled 'Manuscript'.

Guidelines for resubmitting your figure files are available below the reviewer comments at the end of this letter.

We look forward to receiving your revised manuscript.

Kind regards,

Razak M Gyasi, PhD, PD

Academic Editor

PLOS Global Public Health

Journal Requirements:

1. Please provide separate figure files in .tif or .eps format.

For more information about figure files please see our guidelines:  LINK

https://journals.plos.org/globalpublichealth/s/figures

https://journals.plos.org/globalpublichealth/s/figures#loc-file-requirements

If the reviewer comments include a recommendation to cite specific previously published works, please review and evaluate these publications to determine whether they are relevant and should be cited. There is no requirement to cite these works unless the editor has indicated otherwise.

Additional Editor Comments (if provided):

[Note: HTML markup is below. Please do not edit.]

Reviewers' comments:

Reviewer's Responses to Questions

**Comments to the Author**

1. Does this manuscript meet PLOS Global Public Health’s publication criteria?

Reviewer #1: Yes

Reviewer #2: Yes

Reviewer #3: Partly

Reviewer #4: Yes

Reviewer #5: Yes

Reviewer #6: Yes

**********

2. Has the statistical analysis been performed appropriately and rigorously?

Reviewer #1: Yes

Reviewer #2: Yes

Reviewer #3: Yes

Reviewer #4: Yes

Reviewer #5: Yes

Reviewer #6: Yes

**********

3. Have the authors made all data underlying the findings in their manuscript fully available (please refer to the Data Availability Statement at the start of the manuscript PDF file)?

The PLOS Data policy

Reviewer #1: Yes

Reviewer #2: Yes

Reviewer #3: No

Reviewer #4: Yes

Reviewer #5: No

Reviewer #6: No

**********

4. Is the manuscript presented in an intelligible fashion and written in standard English?

Reviewer #1: Yes

Reviewer #2: Yes

Reviewer #3: Yes

Reviewer #4: Yes

Reviewer #5: Yes

Reviewer #6: Yes

**********

Reviewer #1: 1. Title may be more concise

2. In introduction, write details about sociocultural factors affecting UI diagnosis and treatment; What are the healthcare system barriers, and how citations can be reduced

3. In methods, what is the sampling technique; pregnant and women had history of recent childbirth are included or excluded in this study?

4. In table 4, prevalence of UI could be compared across different subgroups

5. In discussion, explain the sociocultural factors more elaborately

Reviewer #2: This is an important paper looking at a pertinent health issue of urinary incontinence affecting women in Uganda.

The paper is generally well written, with a clear methodology and findings on the prevalence of the problem in the study population.

However, the following could make the paper clearer.

I. Introduction

Kindly explain the abbreviation PFH in line 93.

Will be useful to explain the different types of urinary incontinence and their unique causes in the introduction

II. Methodology

Why were these clinics chosen?

Explain the reason for choosing women between 18 and 55 in the methodology

III Discussion

• Kindly summarise the findings clearly in the first paragraph of the discussion

• Authors should speak more to the data in the discussion. For example, clarify the statement below

“This consistency suggests a shared burden shaped by similar population risk profiles and care-seeking

patterns, rather than facility-specific effects.”

• Kindly simplify this to make it clearer and use figures to explain. What higher prevalence figures?What older age structures? What is enrichment for high-risk women?

“Higher clinic-based prevalence estimates reported elsewhere, including studies from Nepal and

Saudi Arabia's are plausibly explained by older age structures, enrichment for high-risk women, or broader recall periods that capture intermittent leakage.

• No explanation of why stress urinary incontinence was the predominant type in the study clinic.

The discussion does not align with the conclusions or recommendations made.I recommend rewriting the discussion to align with the paper's focus.

Reviewer #3: I appreciate you sending me this manuscript. I think it covers an important and underreported topic.

I saw that the manuscript is generally well-organized, the goals are clear, and the statistical analysis is appropriate for the cross-sectional design while I was reading it. I did, however, find a few problems that need to be fixed so that they meet PLOS Global Public Health standards for clarity and transparency. Here is a list of the things that need to be changed:

1) The data that was available did not meet PLOS standards:

PLOS says that all data that supports the findings must be available without any restrictions at the time of publication, with a few rare exceptions.

2) Unreliability of important findings:

I saw some inconsistencies in the reporting of the results. In the abstract section, the distribution of urinary incontinence subtypes was stress 44.7%, urge 30.1%, and mixed 25.2%. In the Results section, however, stress was about 52%, mixed was about 25%, and urgency was about 23%. Also, fix typographical errors in the abstract section, such as changing "stress IU" to "stress UI."

3) How it looks, how it sounds, and how it's put together

The manuscript is written in academic English and is easy to understand, but it needs some grammar and consistency changes because PLOS does not copyedit. Some problems are:

There are fixed terms throughout, like "gynecologic" vs. "gynecological" and "urgency UI" vs. "urge UI."

Make sure that the way you refer to figures and tables is the same (for example, "Fig 1" vs. "Figure 1") and that you report units and time the same way (for example, ">24 h" vs. ">24 hours").

Please make sure that all the tables are clearly labeled and have the right denominator. For example, the headline for table 5 says "Bivariate Logistic Regression of Factors Associated with UI (N=103)," but the number used in the table is 374, which is split into UI present and absent.

Reviewer #4: 1. Title.

The title is appropriate. But the title should be written in title case.

2. Abstract.

Objective: Line 26: State ‘To determine’ instead of Determining

Methods: Line-29: State Kayunga and Jinja Regional Referral Hospitals (RRH)

Result: Line-35: State ‘stress UI’ instead of stress IU

Conclusion: Line-43: State ‘stress UI’ instead of stress urinary incontinence.

3. Introduction section.

Rationale for conducting the study is clear.

Line-87: State pelvic floor health (PFH).

Line 20 & 21: state 'can be' instead of 'will be'.

4. The Methods are described in sufficient details and the ethical concerns has been well described.

Line-99: State ‘from 10th July to 8th October, 2025’ instead of ‘between 10th July and 8th October 2025’

Line-111: Nardos et al., is not required as reference, Vancouver referencing style is enough.

Line 134: Define prolong labor in case of multipara and primipara.

5. Results section.

Line-166: Median age 32 years (interquartile range: 26-39) had not shown in Table 1.

Line-173: Add (N=374) with title of Table 2

Line-175: Add (N=374) with title of Table 3

Line-179: State ‘near similar’ instead of similar

Line-183: State ‘UI’ instead of urinary incontinence and stress UI instead of stress urinary incontinence

Line 184: State ‘Mixed UI’ instead of mixed urinary incontinence

Line 185: State ‘urgency UI’ instead of urgency urinary incontinence

6. Discussion section

Line-226: Write the elaboration of ICIQ-SF

Line-251: State ‘UI’ instead of urinary incontinence and stress UI instead of stress urinary incontinence

Line 252: State ‘urge and mixed UI’ instead of urge and mixed urinary incontinence

Line 256: State ‘stress UI’ instead of stress urinary incontinence

Line 259: State ‘stress UI’ instead of stress urinary incontinence

Line 267: Define NHANES

7. References.

Line-386: Mention the name of the journal in abbreviated form

Line-392: Mention the name of the journal in abbreviated form

Line-395: Mention the name of the journal in abbreviated form

Line-399: Mention the name of the journal in abbreviated form

Line-409: Mention the name of the journal in abbreviated form with volume and issue number

Line-411: Mention the name of the journal in abbreviated form

Line-424: Write the name of the article in Title case and Journal name in correct abbreviated form

Line-427: Mention the name of the journal with volume and issue number.

Line-434: Mention the name of journal in abbreviated form

8. Standard of English for publication.

The English is clear to understand with some incomprehensible parts. The article requires thorough proof-reading before publishing.

Reviewer #5: The title is appropriate. The methodology is okay and the data analysis process is satisfactory. The figures are not understandable. The boxes are disjoint and one cannot understand their connections. Resolution is very poor. It is very blurred and poor. The authors should revise the figures and provide a clear explanation of each figure.

The references should be revised to align with a specific reference style as approved by the journal.

Copy-editing is required to correct all grammatical errors.

Reviewer #6: GENERAL COMMENT:

The study described the prevalence of urinary incontinence and its associated factors among Ugandans using data from two tertiary health institutions in Uganda. The study was well articulated , with adequate data presentations and statistical analyses to support the claims. It was well written in good and concise English language.

SPECIFIC COMMENTS:

Listed below are some observations that may require reviews by the author:

Line 26-17: The Objectives may not necessarily be written separately. It may appear as the last sentence under the "Background" heading. To read: "This study therefore determined......

Lines 62 - 66 may be moved to placed after line 83, in order to make presentation on the prevalence flow along.

Line 98: Can you further describe the features of the two hospitals used for data collection to include number of bed spaces of each hospital, the tier of service, major communities served in terms of tribes, etc, their major specialties and so on..

Line 103: Should read: ...all women that attended...

Line 110: Can you reference the formula used for the sample size calculation?

Line 122: Can the QUID guideline be provided in / as a supporting information? Reader who are not specialists may be curious to know read about the guideline.

Line 136: "Hypertension and diabetes mellitus were assessed by the self-report of a prior clinician’s diagnosis"... How reliable can this data be? Why not extract directly from case notes if available? Or is there any assurance of the reliability of such data? Please revisit this aspect.

Line 142: IS there any criteria for selecting variable <0.20 for multivariate analysis? Is there any statistical provision supporting that?

Lines 157 - 161: This aspect should be part of the methodology, possibly under subject recruitment or data collection.

Line 267: Please give full meaning of NHANES before abbreviations.

Lines 273 - 276: Why not write these aspects out specifically as part of strengths and limitations of the study, amidst other similar observable ones?

Under Discussion - Well articulated and expressed. However, giving a heading for Conclusion and Recommendation may not be necessary. There should be written as paragraphs under the Discussion heading.

FIgure 1 attached is not too clear and understood.

**********

what does this mean?). If published, this will include your full peer review and any attached files.

**Do you want your identity to be public for this peer review?**  If you choose “no”, your identity will remain anonymous but your review may still be made public.

For information about this choice, including consent withdrawal, please see our Privacy Policy

Reviewer #1: No

Reviewer #2: No

Reviewer #3: **Yes:**  Yara Yousef Alfauri

Reviewer #4: **Yes:**  Dr. Nadia Alam

Reviewer #5: **Yes:**  Professor Boluwaji Ade Akinnuwesi

Reviewer #6: **Yes:**  DR. KAZEEM AKINWANDE

**********

[NOTE: If reviewer comments were submitted as an attachment file, they will be attached to this email and accessible via the submission site. Please log into your account, locate the manuscript record, and check for the action link "View Attachments". If this link does not appear, there are no attachment files.]

Attachment Submitted filename: Review of PLOS One.docx

# Author comment: Author response to Decision Letter 1

**DOI:** 10.1371/journal.pgph.0007221.r002

5 Apr 2026

Attachment Submitted filename: Response to Reviewersv2.docx

# Peer review: Decision Letter 1

**DOI:** 10.1371/journal.pgph.0007221.r003

**Authors:** Razak M Gyasi

27 Apr 2026

PGPH-D-25-04011R1

Urinary Incontinence in Ugandan Gynecologic Outpatient Clinics: Prevalence, Clinical Types, and Associated Factors

PLOS Global Public Health

Dear Dr. Kasujja,

Thank you for submitting your manuscript to PLOS Global Public Health. After careful consideration, we feel that it has merit but does not fully meet PLOS Global Public Health’s publication criteria as it currently stands. Therefore, we invite you to submit a revised version of the manuscript that addresses the points raised during the review process.

Please submit your revised manuscript by May 27 2026 11:59PM. If you will need more time than this to complete your revisions, please reply to this message or contact the journal office at globalpubhealth@plos.org. When you're ready to submit your revision, log on to https://www.editorialmanager.com/pgph/ and select the 'Submissions Needing Revision' folder to locate your manuscript file.

Please include the following items when submitting your revised manuscript:

- A letter that responds to each point raised by the editor and reviewer(s). You should upload this letter as a separate file labeled 'Response to Reviewers'.
- A marked-up copy of your manuscript that highlights changes made to the original version. You should upload this as a separate file labeled 'Revised Manuscript with Track Changes'.
- An unmarked version of your revised paper without tracked changes. You should upload this as a separate file labeled 'Manuscript'.

Guidelines for resubmitting your figure files are available below the reviewer comments at the end of this letter.

As the corresponding author, your ORCID iD is verified in the submission system and will appear in the published article. PLOS supports the use of ORCID, and we encourage all coauthors to register for an ORCID iD and use it as well. Please encourage your coauthors to verify their ORCID iD within the submission system before final acceptance, as unverified ORCID iDs will not appear in the published article. *Only*  the individual author can complete the verification step; PLOS staff *cannot*  verify ORCID iDs on behalf of authors.

We look forward to receiving your revised manuscript.

Kind regards,

Professor Razak Gyasi, PhD, PD

Academic Editor

PLOS Global Public Health

Journal Requirements:

If the reviewer comments include a recommendation to cite specific previously published works, please review and evaluate these publications to determine whether they are relevant and should be cited. There is no requirement to cite these works unless the editor has indicated otherwise.

Please review your reference list to ensure that it is complete and correct. If you have cited papers that have been retracted, please include the rationale for doing so in the manuscript text, or remove these references and replace them with relevant current references. Any changes to the reference list should be mentioned in the rebuttal letter that accompanies your revised manuscript. If you need to cite a retracted article, indicate the article’s retracted status in the References list and also include a citation and full reference for the retraction notice.

Additional Editor Comments (if provided):

Dear Authors,

Thanks for revising and improving the quality of your paper. However, Reviewers #1 and #3 have suggested very important minor issues that should be addressed before I can progress your paper. Please, be meticulous in considering these comments and also respond appropriately.

Good luck!

[Note: HTML markup is below. Please do not edit.]

Reviewers' comments:

Reviewer's Responses to Questions

**Comments to the Author**

Reviewer #1: All comments have been addressed

Reviewer #3: All comments have been addressed

Reviewer #4: All comments have been addressed

**********

publication criteria?

Reviewer #1: Yes

Reviewer #3: Partly

Reviewer #4: Yes

**********

3. Has the statistical analysis been performed appropriately and rigorously?

Reviewer #1: Yes

Reviewer #3: Yes

Reviewer #4: Yes

**********

4. Have the authors made all data underlying the findings in their manuscript fully available (please refer to the Data Availability Statement at the start of the manuscript PDF file)?

The PLOS Data policy

Reviewer #1: Yes

Reviewer #3: Yes

Reviewer #4: Yes

**********

5. Is the manuscript presented in an intelligible fashion and written in standard English?

Reviewer #1: Yes

Reviewer #3: Yes

Reviewer #4: Yes

**********

Reviewer #1: Meet my requirements as much as possible

Reviewer #3: This manuscript addresses an important and underrepresented area in global public health, specifically the burden of urinary incontinence among women attending gynecologic outpatient clinics in Uganda. The study is well structured, uses a validated diagnostic tool (QUID), and provides meaningful insights into prevalence, clinical subtypes, and associated factors within a referral-level setting. The findings are relevant and have potential implications for clinical practice and health system strengthening in low- and middle-income countries.

Major comments:

1. Clarification of novelty:

The authors should more clearly articulate what distinguishes this study from existing literature in sub-Saharan Africa. While the setting is relevant, the novelty compared to prior clinic-based studies should be emphasized more explicitly in the Introduction.

2. Limitations and bias:

The limitations section should be expanded. In particular:

* The use of consecutive sampling in referral hospitals introduces selection bias and limits generalizability.

* The cross-sectional design precludes causal inference.

* Reliance on self-reported variables (e.g., comorbidities, obstetric history) may introduce recall bias.

* The use of a symptom-based tool rather than clinical or urodynamic confirmation may lead to misclassification.

3. Confounding factors:

Important potential confounders such as menopausal status are not included and should be acknowledged. Additionally, the role of BMI and other clinical variables should be more critically interpreted.

4. Statistical analysis:

While generally appropriate, the manuscript would benefit from:

* Brief mention of model diagnostics (e.g., goodness-of-fit)

* Discussion of wide confidence intervals for some estimates

* Consideration of potential residual confounding

5. Public health implications:

Given the journal’s scope, the Discussion should further elaborate on how these findings can be translated into practice. For example:

* Feasibility of routine screening in outpatient clinics

* Integration into maternal and reproductive health services

* Potential for task-shifting to non-specialist providers

6. Causal language:

Some statements imply causality despite the cross-sectional design. The authors should ensure consistent use of associative language (e.g., “associated with” rather than “leads to”).

Minor comments:

* Some tables are dense and could be simplified or moved to supplementary materials.

* Minor grammatical and stylistic edits are needed throughout the manuscript.

* Redundant phrasing in the Introduction and Discussion should be reduced.

Conclusion:

Overall, this is a valuable contribution to the literature and is suitable for publication after minor to moderate revisions addressing the points above.

Reviewer #4: Rereview of the article titled “Urinary Incontinence in Ugandan Gynecologic Outpatient Clinics: Prevalence, Clinical Types, and Associated Factors”

1. Title.

The title is appropriate.

2. Abstract.

It is appropriate and corrected.

3. Introduction section.

Line-85: State Kayunga and Jinja Regional Referral Hospitals (RRH)

4. The Methods section

Line-92: State Kayunga and Jinja Regional Referral Hospitals (RRH)

Line-98: State Kayunga and Jinja Regional Referral Hospitals (RRH)

Line-137: State Body mass index (BMI). Because, BMI category is mentioned in Table 5 & 6

5. Results section.

Line-171: State Body mass index (BMI)

6. References.

Mention the DOI of the article with volume and issue number where applicable

**********

what does this mean?). If published, this will include your full peer review and any attached files.

**Do you want your identity to be public for this peer review?**  If you choose “no”, your identity will remain anonymous but your review may still be made public.

For information about this choice, including consent withdrawal, please see our Privacy Policy

Reviewer #1: No

Reviewer #3: No

Reviewer #4: **Yes:**  Nadia Alam

**********

[NOTE: If reviewer comments were submitted as an attachment file, they will be attached to this email and accessible via the submission site. Please log into your account, locate the manuscript record, and check for the action link "View Attachments". If this link does not appear, there are no attachment files.]

Attachment Submitted filename: Rereview PLOS Global Public Health.docx

# Author comment: Author response to Decision Letter 2

**DOI:** 10.1371/journal.pgph.0007221.r004

17 Jun 2026

Attachment Submitted filename: Response to Reviewers.docx

# Peer review: Decision Letter 2

**DOI:** 10.1371/journal.pgph.0007221.r005

**Authors:** Yong Zhang

14 Aug 2026

Urinary Incontinence in Ugandan Gynecologic Outpatient Clinics: Prevalence, Clinical Types, and Associated Factors

PGPH-D-25-04011R2

Dear Dr. Kasujja,

We are pleased to inform you that your manuscript 'Urinary Incontinence in Ugandan Gynecologic Outpatient Clinics: Prevalence, Clinical Types, and Associated Factors' has been provisionally accepted for publication in PLOS Global Public Health.

Before your manuscript can be formally accepted you will need to complete some formatting changes, which you will receive in a follow up email. A member of our team will be in touch with a set of requests.

Please note that your manuscript will not be scheduled for publication until you have made the required changes, so a swift response is appreciated.

IMPORTANT: The editorial review process is now complete. PLOS will only permit corrections to spelling, formatting or significant scientific errors from this point onwards. Requests for major changes, or any which affect the scientific understanding of your work, will cause delays to the publication date of your manuscript.

If your institution or institutions have a press office, please notify them about your upcoming paper to help maximize its impact. If they'll be preparing press materials, please inform our press team as soon as possible -- no later than 48 hours after receiving the formal acceptance. Your manuscript will remain under strict press embargo until 2 pm Eastern Time on the date of publication. For more information, please contact globalpubhealth@plos.org.

Thank you again for supporting Open Access publishing; we are looking forward to publishing your work in PLOS Global Public Health.

Best regards,

Yong Zhang, Ph.d

Academic Editor

PLOS Global Public Health

***********************************************************

Reviewer Comments (if any, and for reference):

Reviewer's Responses to Questions

**Comments to the Author**

Reviewer #3: All comments have been addressed

Reviewer #4: All comments have been addressed

**********

publication criteria?

Reviewer #3: Yes

Reviewer #4: Yes

**********

3. Has the statistical analysis been performed appropriately and rigorously?

Reviewer #3: Yes

Reviewer #4: Yes

**********

4. Have the authors made all data underlying the findings in their manuscript fully available (please refer to the Data Availability Statement at the start of the manuscript PDF file)?

The PLOS Data policy

Reviewer #3: Yes

Reviewer #4: Yes

**********

5. Is the manuscript presented in an intelligible fashion and written in standard English?

Reviewer #3: Yes

Reviewer #4: Yes

**********

Reviewer #3: I am satisfied that the authors have adequately addressed the concerns that were raised in the previous round of review. The revised manuscript is technically sound, ethically conducted, and clearly presented. The conclusions are substantiated by the data presented, and the study design, data collection procedures, and statistical approach are suitable for the stated objectives. The manuscript also includes a Data Availability Statement that is sufficient to confirm that the de-identified participant-level dataset and data dictionary are accessible as supporting information. I have no additional comments and believe that the manuscript is suitable for publication.

Reviewer #4: Review of the revised manuscript titled "Urinary Incontinence in Ugandan Gynecologic Outpatient Clinics: Prevalence, Clinical Types, and Associated Factors"

Comment:

All comments have been carefully addressed, and the manuscript has been revised to the meet the requirements.

**********

what does this mean?). If published, this will include your full peer review and any attached files.

**Do you want your identity to be public for this peer review?**  If you choose “no”, your identity will remain anonymous but your review may still be made public.

For information about this choice, including consent withdrawal, please see our Privacy Policy

Reviewer #3: **Yes:**  Yara Yousef Alfauri

Reviewer #4: **Yes:**  Dr. Nadia Alam

**********
